# The origin of genetic diversity of indigenous cockfighting chickens of Pakistan by analyzing the mtDNA

**DOI:** 10.1016/j.heliyon.2024.e27755

**Published:** 2024-03-16

**Authors:** Sajid Mahmood, Mian Sayed Khan, Zaib Ullah, Raman Akinyanju Lawal, Olivier Hanotte

**Affiliations:** a Department of Zoology, Hazara University, Mansehra, 21300, Pakistan; b Department of Zoology, University of Swabi, Khyber Pakhtunkhwa, Pakistan; c School of Life and Environmental Sciences, Deakin University Waurn Ponds Campus, Victoria 3216, Australia; d School of Life Sciences, University of Nottingham, University Park, Nottingham, UK; e The Jackson Laboratory, 600 Main Street, Bar Harbor 04609, Maine, USA; f Department of Zoology, University of Chakwal, Main Campus, Talagang Road, 48800, Chakwal, Pakistan

**Keywords:** Cockfighting, mtDNA, D-loop, Introgression, Pakistan

## Abstract

In Pakistan, the origin of the indigenous cockfighting chicken (ICC) or gamecock population is unknown. However, it is speculated that this might have been associated with domestication, an event linked to recreational, entertainment (cockfighting), religious or ornamental activities. This study aims to understand the origin and genetic diversity of the ICC population in Pakistan.

A total of 185 ICC population and 10 captive Indian red junglefowl (*Gallus gallus murghi*) were analyzed for genetic diversity indices and phylogenetic reconstruction using a 397 bp of mtDNA D-loop region.

It is reported that a total of 43 haplotypes from 38 polymorphic nucleotide sites. The haplotype and nucleotide diversity are also estimated in the range of 0.643–0.909, and 0.00585–0.01575, respectively. The total genetic diversity within the population was 91.52%. Four mitochondrial haplogroups A, B, C and D were identified by median-joining network analysis, two of them have high percentages, haplogroup D (81.6%) and A (15.1%). Phylogenetic analysis showed that the ICC population of Pakistan and *Gallus gallus murghi* shared haplogroup D. The results of this study showed that sub-haplogroup D17a05, has significantly high haplotype diversity and percentage as compared to previously published studies, this indicated that Pakistan might be one of the centres of domestication for chicken, as it is considered that Southeast Asia is the centre of domestication. Frequencies of Haplogroup A also indicate South-North indices. This research work showed that the indigenous cockfighting chicken population of Pakistan is genetically introgressed from *Gallus gallus murghi*, and significant variations could be attributed to the underlying differences in the geographics, selection pressures, introgression, and regional practices; and multiple origins of cockfighting chickens’ populations around the world which reflected the past trading routes between human communities and civilizations.

## 1 Introduction

In the Indian subcontinent the indigenous cockfighting chicken populations are famous for their vigour, strength, vigilance, forcefulness, alertness, aggressiveness, fighting behaviour and disease resistance ability. Pakistan, one of the centres of origin of agriculture, hosts a large number of indigenous livestock, including village chickens. Seals depicting cockfighting, perhaps as old as 2500 BC have been found at Mohenjo-Daro (Sindh Province) [1]. Previous reports reveal that there are four-seven common varieties of ICC in Pakistan, which are distributed in different agroecological regions. Their geographical disparity has led to the emergence of differences in their physical appearance and several other phenotypic characteristics [2]. The evolutionary process (introgression and interspecies hybridization) shows their role in the adaptation and genetic history of the species [3] for improving the productivity of agricultural plants and livestock, The interspecies hybridization commonly practised [4] occurs between domestic and wild species [5] and is also common in Galliformes and wild birds [[6], [7], [8], [9], [10]]. Moreover, gene flow between Silver Pheasant and Kalij Pheasant was indicated by the analysis of mtDNA and nuclear microsatellites [11]. Additionally, signatures of introgression between wild Gallus species (Grey junglefowl *G. sonneratii*, Ceylon junglefowl *G. lafayettii*, Red junglefowl *G. gallus* and Green junglefowl *G. varius*) and indigenous domestic village chicken were reported [12]. Similarly, genetic introgression was reported in Malagasy chickens among fighting, meat, and commercial lines [13], gamecocks and indigenous chickens in China [14] and domestic chickens were derived from Red junglefowl (*G. gallus spadiceous*) [15].

Generally, chickens including cockfighting (Gamecock) were domesticated in Southeast Asia about 8000 years ago [12] or earlier around 9500 years ago [15], either independently or as diffusion from Southeast Asia. Pakistan also hosted the wild ancestor of a domestic chicken, the red junglefowl, but the status of this wild red junglefowl subspecies in the country remains poorly documented. During the domestication event from Red junglefowl, chickens became admixed with other wild junglefowl species which subsequently improved their genetic diversity [12,16,17]. The chicken may have initially been domesticated for cultural reasons such as religion, decoration, and cockfighting instead of as a food resource [18]. Mitochondrial studies indicate that multiple chicken domestication centres [19] and human influence chickens were dispersed from the Indian subcontinent and South-East Asia [20,21]. *Gallus gallus gallus* and *Gallus gallus spadiceus* were the original ancestors of the Chinese native cockfight chicken breeds [22]. Similarly, Japanese domesticated chickens, including the ornamental varieties and Naganakidori, are derived from the ancestors of the Shamo (cockfighting) in Okinawa [23,24]. The Asian subcontinent has been proposed as the initial centre of origin for the haplogroup D [21,[25], [26], [27]] and the continental Southeast Asia ancestral haplogroup D [28]. Other haplogroups occur in the continent but at a very low frequency [21]. Especially, we would like to address the issue of maternal genetic origin and genetic diversity of cockfighting chickens from Pakistan by the analysis of mtDNA D-loop and comparing with the reference sequences.

## 2 Materials and methods

A total of 185 blood samples of ICC population were collected from seven distinct geographical regions namely: Sindh (n = 33), Punjab (PU) (n = 49), Khyber Pakhtunkhwa (KP) (n = 71), Federally Administered Tribal Area (FATA) (n = 11), Azad Jammu and Kashmir (AJK) (n = 8), Gilgit Baltistan (GB) (n = 3) and samples of *Gallus gallus murghi* (n = 10) from Dhodial Pheasantry, Mansehra. Blood samples were stored in ethylenediaminetetraacetic acid EDTA-containing vacutainer tubes in the freezer at −20 °C and the genomic DNA isolation process was completed by using ammonium acetate precipitation [29] and phenol-chloroform protocols.

Five hundred and forty-nine base pairs of the mtDNA D-loop region were amplified using (5′-AGGACTACGGCTTGAAAAGC-3′, accession number NC_001323) as the forward primer [21] and H547 (5′-ATGTGCCTGACCGAGGAACCAG-3′, accession number AB098668) as the reverse primer [30]. PCR products were purified using NucleoSpin® Gel and polymerase chain reaction, clean-up kit [31]. Purified products were Sanger sequenced from Source Biosciences using AV1F2 (5′-AGGACTACGGCTTGAAAAGC-3′) as the forward primer and H547 (5′-ATGTGCCTGACCGAGGAACCAG-3′) as the reverse primers.

For each sample, two fragments were generated. The forward and reverse primers were trimmed, and the remaining fragments were joined to re-construct a 549bp consensus sequence using CodonCodeAligner version 5.1.3. The fragments of each sequence were further aligned against the reference (accession number AB098668) [30] using Clustal X version 2.1 [32]. Subsequent analyses were restricted to the first 397bp which includes the hypervariable region (HV1) [33]. Seven common haplotypes, representing major haplogroups found in Asian domestic chickens and wild red junglefowl, sequences were collected from the Gene bank database used as reference sequences [19,21] to determine the origins of *Aseel* chickens found in Pakistan within the geographic range of the wild ancestor, the *Gallus gallus murghi*. The median-joining network analysis was based on 43 haplotypes of this study along with nine reference sequences corresponding to the nine haplogroups defined by Ref. [19].

A neighbour-joining (NJ) tree was constructed following 1000 bootstrap replicates using MEGA version 6.0 [34]. A median-joining (MJ) network was constructed using NETWORK 4.6.1.2 [35]. Genetic variations such as the nucleotide diversity, haplotype diversity and the average number of nucleotide differences for each population were determined using DnaSP v5 [36]. The population genetic structure was evaluated by the analysis of molecular variance (AMOVA), among the geographic sites of ICC populations from Pakistan.

## 3 Results

**Fig. 1:**
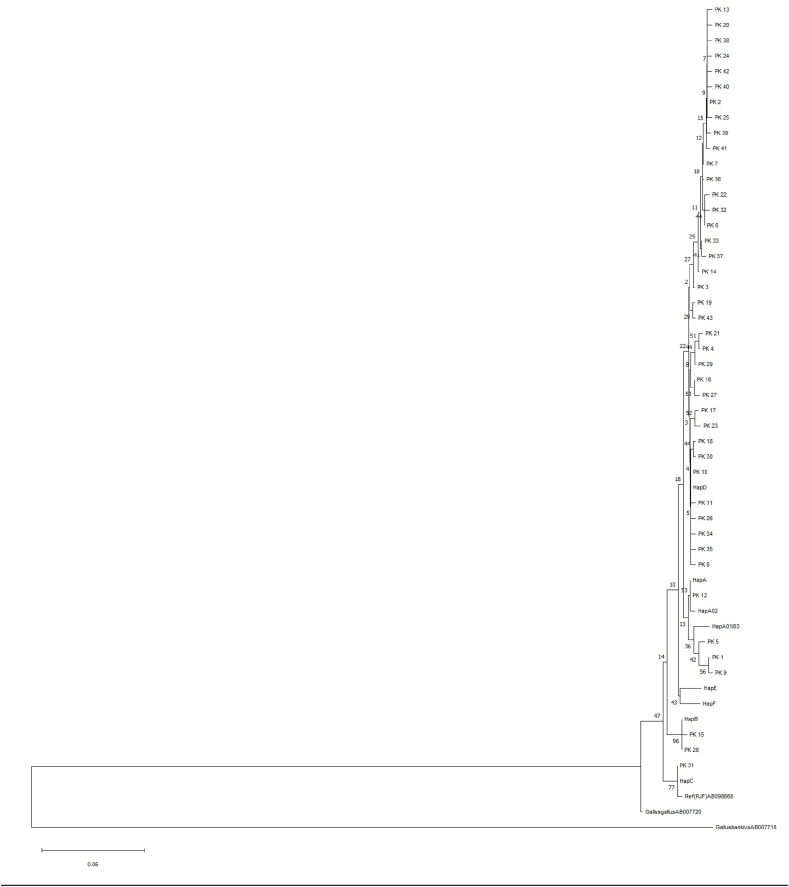
Neighbour-joining tree constructed using 43 haplotypes of ICC of Pakistan. Haplotype of the genus Gallus retrieved from GenBank; *Gallus gallus bankiva* (accession number AB007718) used as an out group and seven haplotypes representing the major chicken mitochondrial DNA clades haplotypes A, B, C, D, E and F as defined by Ref. [19] and Haplotype from *Gallus gallus* (accession number AB098668) are also included in the tree as reference.

By analyzing the mtDNA D-loop 397 bp sequence fragments, we generated 43 haplotypes from 38 polymorphic sites. The haplotypes ranged in cumulative frequency from 0.005 to 0.346. A major haplotype PK_2 with a frequency of 34.6% is present across all populations from geographic regions of Pakistan. Likewise, the second major haplotype PK_10 (represented in *Gallus gallus murghi*) with a frequency of 13.0% was observed. The other two mid-frequent haplotypes PK_5 (9.7%) and PK_6 (8.6%) were distributed in four locations (PU, KP, Sindh and FATA) (Table 1). A major haplotype PK_2 represented in geographic regions and was distributed in ICC populations. Haplotypes PK_2, PK_5 and PK_6 are common to all populations but PK_2 is the most common (Fig. 1).

**Table 1:** Sampling sites, sample size (N), haplotype distribution (F), mutations (Eta) haplotype (Hd) and nucleotide diversities (π), with standard deviations in parentheses, in the ICC population of Pakistan based on mtDNA D-loop sequence comparisons.

Sampling sites	N	F	Eta	Hd	π
Khyber Pakhtunkhwa	71	29	32	0.837 (0.043)	0.01005 (0.00127)
Punjab	49	16	23	0.743 (0.063)	0.01122 (000148)
Sindh	33	10	15	0.847 (0.038)	0.01024 (0.00115)
FATA	11	7	17	0.909 (0.066)	0.01575 (0.00173)
Pheasantry (JF)	10	2	1	0.200 (0.154)	0.00050 (0.00039)
Kashmir	8	4	6	0.643 (0.064)	0.00585 (0.00206)
Gilgit-Baltistan	3	3	5	1 (0.272)	0.0084 (0.00263)
All cockfighting	175	43	38	0.840 (0.024)	0.01074 (0.00071)
All samples	185	43	38	0.847 (0.021)	0.01056 (0.00066)

JF, Jungle Fowl; FATA, Federally Administrated Tribal Area.

**Fig. 2:**
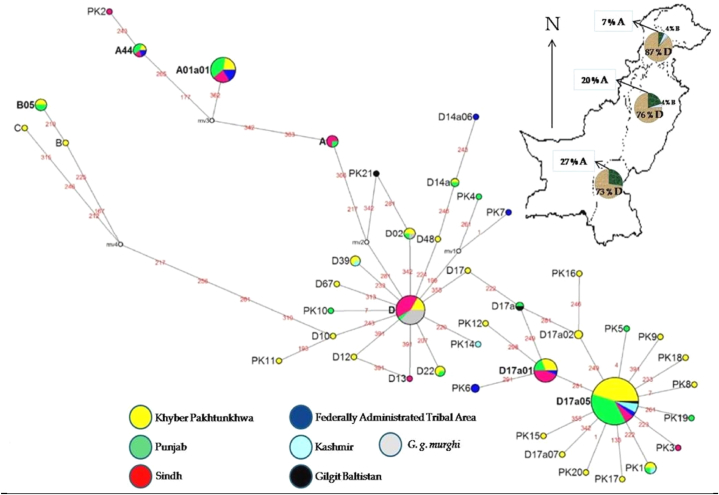
Median-joining network constructed by 43 haplotypes of ICC populations of Pakistan from distinct geographic locations based on the polymorphic sites of the mtDNA D-loop. The area of each circle is proportional to the frequency of the corresponding haplotype. The numbers between the haplotype nodes refer to the positions of nucleotide mutations compared to a reference sequence (Accession number AB098668).

**Fig. 3:**
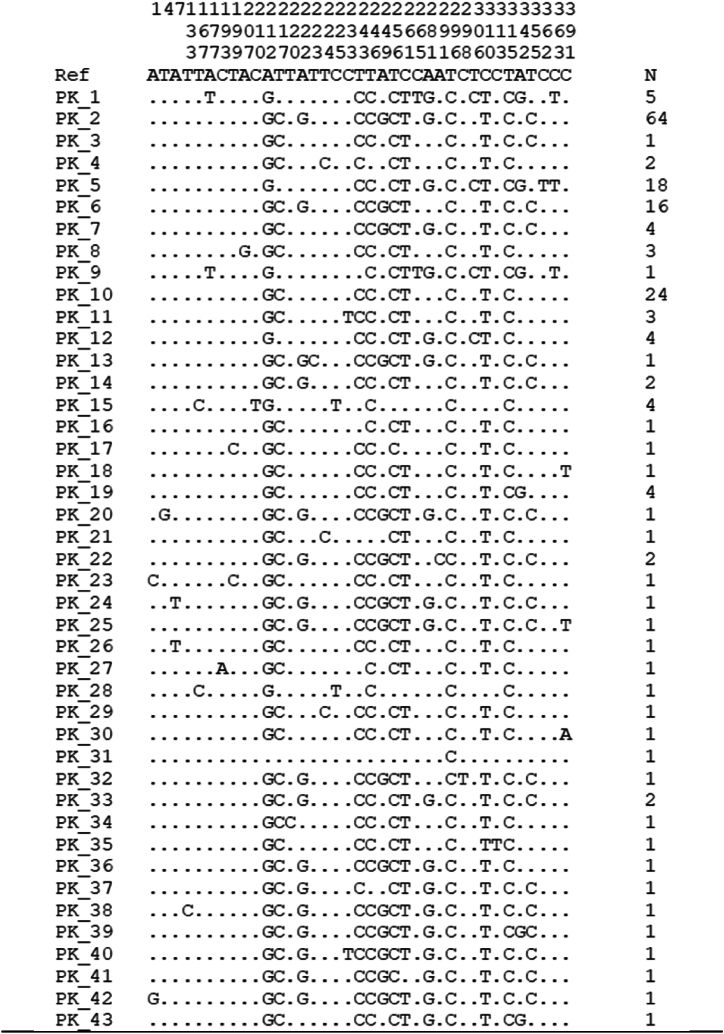
Nucleotide polymorphisms and sequence variations of 43 haplotypes of ICC populations, *Gallus gallus murghi* vertically oriented numbers indicate the sites position and the sequences shown are only the variable sites. Dots (.) indicate identity with the reference sequence (accession number AB098668) [30,38].

Four haplogroups, A, B, C and D, based on shared single nucleotide polymorphism (SNPs) as shown in Fig. 1, Fig. 2, Fig. 3, were identified by Phylogenetic tree and median-joining Network. The dominant clades A and D were represented by ICC populations belonging to distinct geographic regions of Pakistan. Haplogroup D showed two stars as the signature of expansions and represented 81.6% of the population along with three sub-haplogroups D (24 haplotypes), D17a01 (6 haplotypes) and D17a05 (12 haplotypes).

The second large haplogroup A is made up of four haplotypes A, A01a01, A44 and Pk2 - which is reported in 15.1% population. The declining trend in the frequency of haplogroup A was recorded from the South to the Northern part of Pakistan. Percentage frequency of haplogroup A in Southern province Sindh (27%), in PU (20%) and in Northern provinces KP and GB (7%) (Fig. 2).

The remaining (3rd) haplogroup C is represented by a single haplotype found in KP, while haplogroup B is represented by two haplotypes B and B05 - present in PU and KP regions and about 2 % of the population.

Diversity indices for the Pakistani ICC population from various sampling sites ranged about (0.643 ± 0.064) to (0.909 ± 0.066) and (0.0084 ± 0.0026) to (0.01122 ± 0.00014) for haplotypes diversity (Hd) and nucleotide diversity (π) respectively. In the population of Junglefowl least haplotype diversity was reported as compared to the ICC population (Table 2).

**Table 2:** MtDNA D-loop haplotypes and frequency distribution of ICC population in Pakistan.

Haplotypes	PU	KP	Sindh	FATA	Kashmir	GB	Pheasantry	^1^Frequency
PK_1	2	1	1	1	–	–	–	0.027
PK_2	24	28	4	2	5	1	–	0.346
PK_3	1	–	–	–	–	–	–	0.005
PK_4	1	1	–	–	–	–	–	0.011
PK_5	7	4	4	3	–	–	–	0.097
PK_6	3	5	7	1	–	–	–	0.086
PK_7	1	2	–	–	1	–	–	0.022
PK_8	1	2	–	–	–	–	–	0.016
PK_9	–	–	1	–	–	–	–	0.005
PK_10	1	4	10	–	–	–	9	0.130
PK_11	–	1	1	–	1	–	–	0.016
PK_12	1	–	3	–	–	–	–	0.022
PK_13	–	–	1	–	–	–	–	0.005
PK_14	1	–	–	–	–	1	–	0.011
PK_15	2	2	–	–	–	–	–	0.022
PK_16	1	–	–	–	–	–	–	0.005
PK_17	1	–	–	–	–	–	–	0.005
PK_18	–	–	1	–	–	–	–	0.005
PK_19	1	2	–	–	–	–	1	0.022
PK_20	1	–	–	–	–	–	–	0.005
PK_21	–	–	–	1	–	–	–	0.005
PK_22	–	–	–	2	–	–	–	0.011
PK_23	–	–	–	1	–	–	–	0.005
PK_24	–	1	–	–	–	–	–	0.005
PK_25	–	1	–	–	–	–	–	0.005
PK_26	–	1	–	–	–	–	–	0.005
PK_27	–	1	–	–	–	–	–	0.005
PK_28	–	1	–	–	–	–	–	0.005
PK_29	–	1	–	–	–	–	–	0.005
PK_30	–	1	–	–	–	–	–	0.005
PK_31	–	1	–	–	–	–	–	0.005
PK_32	–	1	–	–	–	–	–	0.005
PK_33	–	2	–	–	–	–	–	0.011
PK_34	–	–	–	–	1	–	–	0.005
PK_35	–	1	–	–	–	–	–	0.005
PK_36	–	1	–	–	–	–	–	0.005
PK_37	–	1	–	–	–	–	–	0.005
PK_38	–	1	–	–	–	–	–	0.005
PK_39	–	1	–	–	–	–	–	0.005
PK_40	–	1	–	–	–	–	–	0.005
PK_41	–	1	–	–	–	–	–	0.005
PK_42	–	1	–	–	–	–	–	0.005
PK_43	–	–	–	–	–	1	–	0.005

PU, Punjab; KP, Khyber Pakhtunkhwa; FATA, Federally Administrated Tribal Area; GB, Gilgit Baltistan; PK, Pakistan;^1^The cumulative frequency of the cockfighting chicken population.

Analysis of molecular variance (AMOVA) results showed the total genetic variation within the ICC population was 91.52%, while 8.48% genetic differentiation among the studied population geographically. Low geographical genetic differentiation suggests that the Pakistani ICC population have not been subdivided across the regions, hence this implies that breeding females may have been exchanged (Table 3).

**Table 3:** Analysis of Molecular Variance (AMOVA) among mtDNA D-loop haplotypes of Cockfighting Chickens of Pakistan.

Source of variation	df	Sum of squares	Variance components	Percentage of variation	P-value
Among	6	7.616	0.03612	8.48	aP < 10^−6^
Within the Populations	168	65.470	0.38970	**91.52**	
Total	174	73.086	0.42582		

a *Highly significant*.

## 4 Discussion

Our findings are supported by several previous studies, that suggest the Indian subcontinent is the initial centre of origin for haplotype D [21,25,26]. The haplogroup D is also reported in the gamecocks of Indonesia, India and Japan [19,37]. Overall, haplogroup D was observed in 81.6% of the population, and sub-haplogroups (D17a05, D, and D17a01) were frequently observed in 65.9% of the populations of the three sub-haplogroups, D17a05 was recorded as genetically diverse haplogroup, having 12 different haplotypes, and consists of 49.6% of haplogroup D. Thus, the high genetic diversity of haplogroup D shows that, in the past, Pakistan might have been one of the centres for chicken domestication while researchers believed that the main centre of domestication was Southeast Asia [28,37].

The phylogenetic tree also showed that haplotypes were clustered into four haplogroups (A, B, C, D) out of a total of nine previously identified haplogroups in Asian domestic chickens by Liu et al. (2006), out of 36 haplotypes (83.7%) were clustered into haplogroup D followed by haplogroup A (4 haplotypes), B (2 haplotypes), and C (1 haplotype). Haplogroup A, on the other hand, was the least diverse containing only four haplotypes. Haplogroup A, although unexpectedly, revealed a South to North declining trend in Pakistan. Arguably, this could corroborate the previous idea of geographical and anthropological contact between Africa and South Asia [21]. In other words, haplogroup A has been recently introduced in Pakistan from South East Asia via Africa through recent past trading [26,27,37,38].

Overall haplotype and nucleotide diversity in the population was similar to that of Sri Lankan [39] and Indian [40] domestic chickens whereas low nucleotide diversity in the population was in agreement with the previous reports from Chinese and Eurasian [19] and domestic chicken population from Japan [37]. Likewise, lower diversity measures from African chicken populations were recorded in a previous study [26]. Over results showed, that mtDNA (HV1) D-loop region was highly variable in the studied population and observed a mean number of nucleotide differences (K) among haplotypes of (4.19 ± 1.2). Our findings suggest comparable estimates of genetic diversity (0.840) with India (0.66) [41] and Bangladesh (0.923) [42], however, our results showed significant differences with other such estimates for countries like Japan (0.0016) [37] and China (0.045) [43]. Sri Lanka, on the other hand, revealed a slightly higher estimate of genetic diversity (0.947) than our findings [39]. The observed homogeneity between our findings and those reported from India and Bangladesh could partially be attributed to the factors like close geographic proximity, socio-cultural similarity, and frequent human migration for making their livelihoods through agriculture and livestock exchange including chickens. The observed disharmony between our findings and those reported from China and Japan could be due to the marked geo-political and socio-cultural differences between Pakistan and the later ones.

## 5 Conclusions

A total of 43 haplotypes defined by the 38 polymorphic sites among the 185 sequences were identified. Overall, high genetic diversity was reported in the cockfighting chicken population. Pakistan may be the possible centre for the domestication of cockfighting chickens. Cockfighting chickens have had a substantial influence on the dispersal of the species throughout the world providing a proxy for the understanding of past trading relationships between human communities and civilizations.

## Ethical approval certificate

On June 25, 2022, the Institutional Ethical Committee of Hazara University, Mansehra, granted ethical approval for the article titled “The genetic diversity and origin of indigenous cockfighting chickens of Pakistan by using mtDNA," authored by Dr. Sajid Mahmood and co-authors. This research, conducted under the supervision of Prof. Dr. Mian Sayed Khan (Pakistan) and Olivier Hanotte (UK), has been authorized for data collection and laboratory work.

The Committee reviewed the proposal and found no ethical concerns that could potentially impact the community. In fact, they recommended proceeding with the research, emphasizing the importance of obtaining proper permissions from chicken owners. This ethical approval certificate affirms that the study adheres to ethical standards and guidelines established by Hazara University's Institutional Ethical Committee.

## Availability of data and materials

Data from this article is available for any sort of publicity after publication.

## Funding

This study was supported by the 10.13039/501100000837University of Nottingham, UK and the 10.13039/501100010221Higher Education Commission of Pakistan for financial support.

## CRediT authorship contribution statement

**Sajid Mahmood:** Writing – original draft, Visualization, Software, Methodology, Data curation, Conceptualization. **Mian Sayed Khan:** Validation, Supervision, Formal analysis. **Zaib Ullah:** Writing – review & editing, Writing – original draft, Visualization, Software, Methodology, Data curation. **Raman Akinyanju Lawal:** Visualization, Software, Resources, Project administration, Funding acquisition, Formal analysis, Conceptualization. **Olivier Hanotte:** Validation, Supervision, Software, Resources, Methodology, Funding acquisition, Formal analysis.

## Declaration of competing interest

The authors declare the following financial interests/personal relationships which may be considered as potential competing interests:Sajid Mahmood reports financial support and travel were provided by 10.13039/501100010221Higher Education Commission of Pakistan. Zaib Ullah reports a relationship with Deakin University Faculty of Science Engineering and Built Environment that includes: employment. If there are other authors, they declare that they have no known competing financial interests or personal relationships that could have appeared to influence the work reported in this paper.
